# Incomplete Susac’s Syndrome: A Case Report and Literature Review

**DOI:** 10.7759/cureus.27903

**Published:** 2022-08-11

**Authors:** Anam Ahmad, Farina Tariq, Muhammad Zaheer

**Affiliations:** 1 Internal Medicine, St. Luke's Hospital, Chesterfield, USA; 2 Internal Medicine, Quaid-e-Azam Medical College, Bhawalpur, PAK; 3 Rheumatology, St. Luke's University Health Network, St. Louis, USA

**Keywords:** autoimmune, vasculitis, hearing loss, retinal artery occlusion, susac syndrome

## Abstract

Susac syndrome (SS) is rare microangiopathy of unclear etiology involving arteries of the brain, cochlea, and retina, affecting mainly middle-aged women. The diagnosis of Susac syndrome is based on a clinical evaluation of the signs and symptoms supported by imaging modalities. Immunosuppressants are the first-line treatment. Our patient is a 46-year-old man who was evaluated for right-sided visual loss and bilateral hearing loss. His ophthalmic examination revealed retinal artery occlusion. He showed a good response to rituximab and his vision remained stable. Our case is particularly unique as it shows an incomplete Susac syndrome involving the cochlea and retina only. This paper aims to increase awareness about the disease's symptoms, treatment, and prognosis.

## Introduction

Susac’s syndrome is rare microangiopathy with an unclear origin, though to be autoimmune, affecting the brain, cochlea, and retina [1]. The syndrome is characterized by a clinical triad of encephalopathy involving corpus callosum, hearing loss, and visual disturbances. The disorder was first described in 1979 by John O. Susac [2]. It affects middle-aged women more than men, with a female/male ratio of 3:1 [3]. Only a few hundred cases have been reported so far. The natural history of the disease is unknown, but early diagnosis and aggressive treatment have been shown to slow the progression of the disease. Our case represents an incomplete Susac’s syndrome manifesting two out of three classic clinical features.

## Case presentation

Our patient is a 46-year-old man who presented to the hospital with sudden-onset visual loss in the right inferior quadrant of the visual field. He didn't report any other symptoms. His past medical history was significant for two similar episodes in his left eye a few years ago due to branch retinal artery occlusion (BRAO) of unclear etiology, which led to permanent left-sided visual field defects. He also has a history of irreversible sensorineural hearing loss in both ears and underwent hearing aid placement in the right ear a year before presentation. He was evaluated by ophthalmology on admission and was found to have branch retinal artery occlusion of the right eye. His labs were mostly unremarkable, including complete blood count (CBC), comprehensive metabolic panel (CMP), anti-nuclear antigen (ANA), extractable nuclear antigens (ENA) profile, antineutrophilic cytoplasmic antibodies (ANCA) antibodies, inflammatory markers, and thrombophilia workup including antiphospholipid antibodies panel, protein C and S deficiency and factor V Leiden deficiency. He had extensive neuroimaging, including resonant magnetic imaging (MRI) of the brain and neck, computed tomographic angiography of the head and neck, transthoracic echocardiogram, and Holter monitoring which were unremarkable. Considering the history of recurrent visual loss due to branch retinal artery occlusion (BRAO), sensorineural hearing loss, and negative workup for other systemic causes, Susac's syndrome was considered a likely etiology for his clinical presentation. He didn't have a history of encephalopathy, so he likely had incomplete Susac's syndrome. He was started on oral prednisone at a dose of 1 mg/kg daily and IV rituximab for induction to prevent recurrent disease that could lead to permanent blindness. Prednisone was later tapered off. He was later switched to mycophenolate mofetil for maintenance therapy. He is currently being followed in the clinic for over six months with no further episodes of hearing or visual loss, and his vision has remained stable to date.

## Discussion

Susac’s Syndrome is a rare occlusive vasculopathy involving the brain, cochlea, and retinal arteries. The exact mechanism is unknown, but it is thought to be autoimmune-mediated endotheliopathy causing microinfarcts. Studies have shown the presence of anti-endothelial cell antibodies in approximately 25% of patients contributing to the pathophysiology [4].

The classic triad is present only in 15% of the patients at the initial presentation. The most common symptom present in 75% of patients is encephalopathy, including the corpus callosum, characterized by recurrent headaches, cognitive impairment, personality changes, confusion, and motor disturbances. The second most common symptom present in 50% of patients is visual disturbances, including scotomas, visual field defects, and reduced visual acuity. Sensorineural hearing loss is frequently associated with vertigo and tinnitus [1].

The diagnosis of Susac’s Syndrome is mostly clinically supported by imaging modalities. Patients with CNS involvement have specific brain MRI findings. Multifocal snowball-like lesions of the corpus callosum and string pearl appearance on T2 weighted images are characteristic of Susac’s Syndrome [5-7]. These lesions help to differentiate Susac’s Syndrome from other demyelinating conditions. In our patient, typical findings of MRI were not present; he had incomplete Susac syndrome, lacking the typical triad. 

The fundoscopic and fluorescein retinal angiographic studies typically show retinal artery occlusion, arterial arrowing, and small punctate plaques. In a case series, about 68% of the patients showed patchy thinning of the inner retina while the outer retina remained normal [8,9]. The patients can have bilateral sensorineural hearing loss on an audiogram. 

Susac’s Syndrome can be monophasic, polyphasic, or chronic continuous. Monophasic is limited to one to two years. Polyphasic is relapsing and remitting, where the time between relapses can vary from months to several years. In chronic continuous form, symptoms continue without significant remission [10]. It should be differentiated from other inflammatory and demyelinating diseases of the CNS, like multiple sclerosis. Invasive procedures, including brain biopsy and brain angiography, are not usually required for the diagnosis.

Treatment is based mainly on expert opinion and case series due to the rarity of the disease. Immunosuppressive agents are the mainstay of treatment. High-dose steroids, e.g., 1 mg/kg prednisone is typically the first-line therapy. Immunosuppressive agents like azathioprine, mycophenolate mofetil, and cyclosporine are used as steroid-sparing agents and can be started early in the treatment course. In the acute phase, plasma exchange and IVIG have also been used. Rituximab and Infliximab have also been found to be efficacious [11, 12]. Our patient was on a tapering dose of oral steroids at the time of presentation and was switched to Cellcept. 

Prognosis varies depending on the severity of the symptoms and the type of clinical course. Patients can have significant neurological deficits, including gait disturbances and dementia. Severe vision impairment is less frequent, while patients with hearing loss can benefit from cochlear implants [13]. Very few cases have been reported where people have died of complications of Susac’s Syndrome. Although patients can develop disabilities, life expectancy does not differ significantly.

## Conclusions

Susac syndrome is a clinical diagnosis characterized by a triad of encephalopathy, branch retinal artery occlusion, and hearing loss supported by specific brain MRI and fundoscopic findings. We should be aware that the complete clinical triad is present in very few patients at the time of diagnosis, as our patient has an incomplete triad. Due to the progressive nature of the disease, prompt diagnosis and aggressive treatment with immunosuppressives are required to avoid neurological sequelae. 
